# On estimating a constrained bivariate random effects model for meta-analysis of test accuracy studies

**DOI:** 10.1177/09622802211065157

**Published:** 2022-01-07

**Authors:** Mohammed Baragilly, Brian Harvey Willis

**Affiliations:** 1 Department of Mathematics, Insurance and Applied Statistics, 120637 Helwan University, Egypt; 2 Institute of Applied Health Research, 1724 University of Birmingham, UK

**Keywords:** Bivariate model, penalised likelihood, penalty method, meta-analysis, random effects, diagnostic accuracy

## Abstract

Tailored meta-analysis uses setting-specific knowledge for the test positive rate and disease prevalence to constrain the possible values for a test's sensitivity and specificity. The constrained region is used to select those studies relevant to the setting for meta-analysis using an unconstrained bivariate random effects model (BRM). However, sometimes there may be no studies to aggregate, or the summary estimate may lie outside the plausible or “applicable” region. Potentially these shortcomings may be overcome by incorporating the constraints in the BRM to produce a constrained model. Using a penalised likelihood approach we developed an optimisation algorithm based on co-ordinate ascent and Newton-Raphson iteration to fit a constrained bivariate random effects model (CBRM) for meta-analysis. Using numerical examples based on simulation studies and real datasets we compared its performance with the BRM in terms of bias, mean squared error and coverage probability. We also determined the ‘closeness’ of the estimates to their true values using the Euclidian and Mahalanobis distances. The CBRM produced estimates which in the majority of cases had lower absolute mean bias and greater coverage probability than the BRM. The estimated sensitivities and specificity for the CBRM were, in general, closer to the true values than the BRM. For the two real datasets, the CBRM produced estimates which were in the applicable region in contrast to the BRM. When combining setting-specific data with test accuracy meta-analysis, a constrained model is more likely to yield a plausible estimate for the sensitivity and specificity in the practice setting than an unconstrained model.

## 1 Introduction

Tailored meta-analysis^1,2^ has been introduced recently as a method to find a plausible estimate for a diagnostic test's performance in a specific clinical setting. Using routine data on the test positive rate, *r* and disease prevalence, *p* from the practice setting of interest an applicable region in receiver operating characteristic (ROC) space may be derived for the purpose of study selection.^1^ Thus, local knowledge for the test in the practice enables a bounded region of plausible sensitivity-specificity pairs in ROC space to be drawn. This may be used to inform study selection by testing the compatibility of individual studies in terms of the reported sensitivity and specificity with those plausible for the setting of interest In general, once the studies have been selected for tailored meta-analysis, they are aggregated using the bivariate random-effects meta-analysis model as in the case of conventional meta-analysis.

However, tailored meta-analysis is not without shortcomings. In some cases, after ensuring compatibility with the applicable region, there may be too few studies to aggregate and even when there are enough studies it does not guarantee the summary estimate will be in the applicable region. Furthermore, a key feature of tailored meta-analysis is the imposing of constraints on a test's performance in order to define a bounded region in ROC space, yet these constraints are not incorporated in the fitting of the bivariate random-effects meta-analysis model.

Here we propose a constrained model in which the parameters to the bivariate random effects meta-analysis model are estimated as usual but subject to the constraints on the sensitivity and specificity as defined by a bounded region in ROC space. The model requires a different form of optimization and incorporates the Lagrangian in order to manage the constraints. As with the unconstrained bivariate random effects model (BRM), there is no closed-form to the likelihood function,^3^ so no analytical solution to the Maximum Likelihood Estimate (MLE) exists, and numerical solutions are required.

Recently, it has been demonstrated that the BRM may be optimized using bespoke Newton-Raphson (NR) algorithms.^3–5^ The algorithms demonstrated favourable performance characteristics^3^ when compared with the generic *glmer* function in the *lme4* package in R.^6^ For the constrained bivariate random effects model (CBRM) we propose a bespoke maximum likelihood algorithm based on NR iterations that optimizes the objective function using a coordinate ascent approach.

The importance of having good initial values for an NR algorithm is formally described by Kantorovich's theorem^7^ and to this end the initial values for the five parameters are derived from analytical estimates.^8,9^ The CBRM is optimized using the penalty method.^10^ This involves adding a penalty function, which penalises any violation of the constraints, to the objective function.^10^ Thus, the constrained optimization problem is replaced by a sequence of unconstrained sub-problems where the solutions of the unconstrained problems ideally converge to the solution of the original constrained problem.

The impetus behind using a constrained model is to provide summary estimates for the test that are more specific to clinical practice than those provided by conventional meta-analysis. Although this seems plausible, it is important to establish this definitively and determine to what extent a constrained model in meta-analysis is better than conventional meta-analysis.

We develop this in the following sections. In section 2, we briefly describe the basis for deriving a constrained region in ROC space. Derivation of the constrained bivariate random-effects meta-analysis model is introduced in section 3. In section 4, the penalty-based algorithm is developed for the CBRM. In section 5, the CBRM and the BRM are compared using a simulation study and applying them to two real datasets from the literature. In section 6, we end with the discussion and conclusion.

## 2 Defining a constrained region in ROC space for a test

The logic which underpins the constrained region for a test's performance has been previously described in detail^1,2^ and is only outlined here. The accuracy of a test is usually established by comparing its classification with a reference standard. If *s* is the sensitivity and *f* is the false positive rate, where f=1− specificity, then for the test to be clinically useful, *s* ≥ *f*. At a particular threshold, the classifications are usually compared using a 2 × 2 contingency table and all elements of the table are non-negative. This latter observation leads to *s ≥ r* and *f ≤ r*. Another important deduction follows from the definitions of the *s, f, r* and *p* - knowing any three of these allows the fourth to be obtained. Also, from the definitions we have 0 *≤ s, f, r*, *p ≤* 1.

A consequence of these equalities and inequalities is that local knowledge of *r* and *p* allows deductions to be made on what is permissible for the sensitivity and specificity. This is the basis for the constrained region in ROC space. The size of the region is dependent on the uncertainty in the estimates for *r* and *p*, and in general, the wider the confidence intervals the larger the region. Finally, to ensure the confidence intervals for *r* and *p* contain the true values, a minimum 99% confidence interval is usually chosen.^1,11^ This also means that the probability that the constrained region contains the true sensitivity and specificity for the test in the setting is greater than 99%.

## 3 Derivation of the constrained bivariate random-effects meta-analysis model

Similar to the BRM, the CBRM assumes a bivariate normal distribution for the logits of the sensitivity and specificity between studies. In addition, the model uses independent binomial distributions for the true positives, and true negatives within each study.^12^ So the model is of the form:

(1) $$\left(\begin{array}{c} \alpha_{i}\\ \beta_{i} \end{array}\right)∼N\left(\left(\begin{array}{c} \alpha \\ \beta \end{array}\right),\;\;\left(\begin{array}{cc} \sigma_{a}^{2} & \rho \sigma_{a}\sigma_{b}\\ \rho \sigma_{a}\sigma_{b} & \sigma_{b}^{2} \end{array}\right)\right)\;$$

(2) $$TP_{i}|P_{a,i}∼Binomial(n_{a,i},P_{a,i})$$

(3) $$\;\;\;TN_{i}|P_{b,i}∼Binomial(n_{b,i},P_{b,i})\;$$

where $P_{a,i}$ and $P_{b,i}$ represent the ith study-level parameters for the sensitivity and specificity respectively, and $\alpha_{i}=logit(P_{a,i})$ and $\beta_{i}=logit(P_{b,i})$ . Furthermore, α and $\sigma_{a}^{2}$ are the mean and variance for the logit sensitivities, β and $\sigma_{b}^{2}$ are the mean and variance for the logit specificities, and ρ is the correlation between $\alpha_{i}$ and $\beta_{i}$ across studies respectively. For the binomial distributions, $TP_{i}$ , $TN_{i}$ , $n_{a,i}$ and $n_{b,i}$ are the number of true positives, true negatives, diseased, and non-diseased for the ith study respectively.

Thus the log-likelihood function of the bivariate generalized linear mixed effect model may be written as:

(4) $$\begin{array}{rl} l(\alpha ,\beta ,\sigma_{a}^{2},\sigma_{b}^{2},\rho )= & \log\;\overset{k}{\underset{i=1}{\prod }}\;p_{r}(TP_{i},TN_{i}|n_{a,i},n_{b,i})=\overset{k}{\underset{i=1}{\sum }}\;\log\;p_{r}(TP_{i},TN_{i}|n_{a,i},n_{b,i})\\ & =\overset{k}{\underset{i=1}{\sum }}\;\log\;\int \int Bin(TP_{i}|n_{a,i};P_{a,i})Bin(TN_{i}|n_{b,i};P_{b,i})\phi (P_{a,i},P_{b,i};\alpha ,\beta ,\sigma_{a}^{2},\sigma_{b}^{2},\rho )\;dP_{a,i}dP_{b,i} \end{array}$$

where $Bin(TP_{i}|n_{a,i};P_{a,i})$ is the binomial probability mass function for the true positives, $Bin(TN_{i}|n_{b,i};P_{b,i})$ is the binomial probability mass function for the true negatives, and $\phi =\phi (P_{a,i},P_{b,i};\alpha ,\beta ,\sigma_{a}^{2},\sigma_{b}^{2},\rho )$ is the bivariate logit normal probability density function for the logits of the sensitivity and specificity between studies.

The adaptive multidimensional integration algorithms described in Genz and Malik^13^ and Berntsen et al.^14^ is used here to compute the double integration over the random effects in the log-likelihood function above, by using the R function *adaptIntegrate* within the package *cubature*.^15^ So, in order to estimate the five parameters in the CBRM, we need to maximize the log-likelihood function defined in (4), subject to the constraints outlined in section 2 and defined algebraically below. The penalty methods can be used to solve a sequence of unconstrained nonlinear optimization problems whose solutions converge on the solution to the original constrained problem. Here we implement one of the most commonly used penalty functions, the quadratic-loss function.

Let $\Theta =(\alpha ,\;\beta ,\sigma_{a}^{2},\sigma_{b}^{2},\rho )$ , and the log-likelihood function, $l(\alpha ,\beta ,\sigma_{a}^{2},\sigma_{b}^{2},\rho )$ for the bivariate generalized linear mixed effects model is l(Θ) , in order to get the maximum likelihood function of the CBRM, we need to solve the constrained optimization problem:

(5) $$\underset{\Theta }{\arg\;\;\max}\;l(\Theta )\;\;\;\;\;\;\;\mathrm{subject}\;\mathrm{to}\;\;g_{j}(\Theta )\geq 0,\;\;j\in T\;$$

where l(Θ) is the objective function, $g_{j}$ are the inequality constraints and T is a finite set of indices. The inequality constraints are defined as:

$$\begin{array}{rl} g_{1}(\Theta ) & :\;\overset{\prime }{\alpha }+\;\overset{\prime }{\beta }-1\;\geq 0\\ g_{2}(\Theta ) & :\;r_{u}+\;\overset{\prime }{\beta }-1\;\geq 0\\ g_{3}(\Theta ) & :\;\overset{\prime }{\alpha }-\;r_{l}\;\geq 0\\ g_{4}(\Theta ) & :\;\frac{r_{u}}{\;p_{l}}\;-\;\frac{\left(1-p_{l})(1-\overset{\prime }{\beta }\right)}{\;p_{l}}\;-\;\overset{\prime }{\alpha }\geq 0\\ g_{5}(\Theta ) & :\;\overset{\prime }{\alpha }-\;\frac{r_{l}}{\;p_{u}}\;+\;\frac{\left(1-p_{u})(1-\overset{\prime }{\beta }\right)}{\;p_{u}}\geq 0\\ g_{6}(\Theta ) & :\;\overset{\prime }{\alpha }\geq 0\\ g_{7}(\Theta ) & :\;1-\;\overset{\prime }{\alpha }\geq 0\\ g_{8}(\Theta ) & :\;\overset{\prime }{\beta }\geq 0\\ g_{9}(\Theta ) & :\;1-\overset{\prime }{\beta }\geq 0 \end{array}$$

where $\overset{\prime }{\alpha }=logit^{-1}(\alpha )$ , $\overset{\prime }{\beta }=logit^{-1}(\beta )$ , and $\left[r_{l},r_{u}\right]$ and $[p_{l},p_{u}]\;$ are the confidence intervals for *r* and *p* respectively. Using the quadratic penalty method, the constrained optimization problem can be solved by maximizing the penalty function φ(θ,δ) , where δ is the penalty parameter in the Lagrangian function, and the constraints are represented by terms added to the objective function:

(6) $$\varphi (\Theta ,\delta )=\;l(\Theta )-\;\delta \overset{9}{\underset{\;j=1}{\sum }}\;[\min(0,\;g_{j}(\Theta )){]}^{2}$$

where $g_{j}(\Theta )$ is the jth exterior penalty function while δ is the penalty coefficient. So, for the kth iteration, the penalty function can be expressed as:

(7) $$\varphi_{k}(\Theta ,\delta_{k})=\;l(\Theta )-\;\delta_{k}\overset{9}{\underset{\;j=1}{\sum }}\;[\min(0,\;g_{j}(\Theta )){]}^{2}$$

Thus, based on Equation (7) we may use the following pseudocode algorithm to estimate the solution to the CBRM:

### Algorithm 3.1 (penalty method for the CBRM):

*Initialization*: set $\delta_{0}$ = 1, ε→0 , and a starting point $\Theta_{0}$ *;*

**for** k=1,2,…

  *Maximization*: Find an approximate maximizer $\Theta_{k}$ of $\varphi_{k}(\Theta )$ , starting at $\Theta_{k-1}$

  *Convergence*: Terminating when $|\varphi_{k}(\Theta_{k})-\varphi_{k-1}(\Theta_{k-1})|\leq \varepsilon$ ;

  **if** final convergence test satisfied

    **stop** with approximate solution $\Theta_{k}$ ;

  **end** (**if**)

  *Updating*: Set new penalty parameter $\delta_{k+1}$ = 10 $\delta_{k}$ ;

    Set new starting point to $\Theta_{k}$ ;

**end** (**for**)

The maximization step is an important element of the algorithm and this is dealt with in more detail in the next section. Note between iterations we increase $\delta_{k}$ by a factor of 10 to ensure a large penalty. This limits the risk of solutions moving away from the feasible region between iterations.

## 4 Penalty-based co-ordinate ascent algorithm for the CBRM

Recently, NR based algorithms have been reported for estimating the parameters in the BRM.^3^ However, the algorithms may be computationally intensive and may, without appropriate initial values, lead to biased results in the final estimate due to convergence on local maxima.^16^

Although the initial values may be derived using numerical methods, these are most efficiently estimated using analytical methods. Thus we may use analytical univariate weighted estimates for the initial values for α and β where the weights are based on the Hedges and Olkin (HO) estimator^9^ for the between-study variance. An initial value for the correlation may be obtained using the Pearson correlation coefficient to estimate the correlation between the logits of the sensitivities and specificities over the studies. This approach has been validated by simulation studies and provides accurate initial values when optimising the BRM using Newton-Raphson based algorithms.^8^

The co-ordinate ascent algorithm used here is also based on NR iteration. It starts with the initial estimates described, and then updates them repeatedly. At each iteration we start with estimating a single parameter whilst the other parameters remain fixed to their last estimated value. The value of the parameter that maximises the log-likelihood function along its axis is then chosen. The algorithm cycles through the other parameters until at the end of the iteration all five parameters have been updated.

Thus, we start with the initial estimate ${\hat{\Theta }}_{0}=({\hat{\alpha }}_{0},\;{\hat{\beta }}_{0},\;\hat{\sigma }{{}_{a}^{2}}_{0},\hat{\sigma }{{}_{b}^{2}}_{0},{\hat{\rho }}_{0}{)}^{T}$ and for the ith iteration, we update ${\hat{\rho }}_{i-1}$ by fixing the other estimated parameters $\left({\hat{\alpha }}_{i-1},\;{\hat{\beta }}_{i-1},\;\hat{\sigma }{{}_{a}^{2}}_{i-1},\hat{\sigma }{{}_{b}^{2}}_{i-1}\right)$ and maximizing $l(\rho ;\;{\hat{\alpha }}_{i-1},\;{\hat{\beta }}_{i-1},\;\hat{\sigma }{{}_{a}^{2}}_{i-1},\hat{\sigma }{{}_{b}^{2}}_{i-1})$ with respect to ρ , to get the updated estimate, $\;{\hat{\rho }}_{i}$ . Now, the next parameter ${{\hat{\sigma }}_{a}^{2}}_{i-1}$ is updated to $\hat{\sigma }a_{i}^{2}$ by maximizing $l(\sigma_{a}^{2};\;{\hat{\alpha }}_{i-1},\;{\hat{\beta }}_{i-1},\hat{\sigma }{{}_{b}^{2}}_{i-1},{\hat{\rho }}_{i})$ with respect to $\sigma_{a}^{2}$ . Similarly, ${{\hat{\sigma }}_{b}^{2}}_{i-1}$ is updated to $\hat{\sigma }b_{i}^{2}$ by maximizing $l(\sigma_{b}^{2};\;{\hat{\alpha }}_{i-1},\;{\hat{\beta }}_{i-1},\;\hat{\sigma }{{}_{a}^{2}}_{i},{\hat{\rho }}_{i})$ and so on. Hence, at the end of the iteration we have ${\hat{\Theta }}_{i}\;=\;({\hat{\alpha }}_{i},\;{\hat{\beta }}_{i},\;\hat{\sigma }{{}_{a}^{2}}_{i},\hat{\sigma }{{}_{b}^{2}}_{i},{\hat{\rho }}_{i}{)}^{T}$ .

Numerically, we can estimate each parameter separately using the NR method in the ith iteration by following the next steps:

1. Get the first and second derivatives of the log likelihood function *l* w.r.t the underlying parameter to be estimated, lets say ρ , so find $\frac{\partial l\;}{\partial \rho }$ *,* $\frac{\partial^{2}l}{\partial \rho^{2}}$ at the value $\;{\hat{\rho }}_{i}$ .
2. The ith estimation is given by:
$${\hat{\rho }}_{i}=\;{\hat{\rho }}_{i-1}\;-\;\left[\frac{\partial l\;}{\partial \rho }/\frac{\partial^{2}l}{\partial \rho^{2}}\;{|}_{\rho ={\hat{\rho }}_{i-1}}\right]\;$$
3. Consider ${\hat{\rho }}_{i}$ as the MLE, and stop the algorithm if: $|{\hat{\rho }}_{i}-{\hat{\rho }}_{i-1}|\;<\;\varepsilon$ , where ε→0 , otherwise repeat steps 1 and 2 after increasing *i* by 1.

As the coordinate ascent algorithm is based on NR, the theoretical importance of choosing good initial start values to ensure convergence has been previously described [7]. Based on the HO initial start, the following penalty algorithm may be used to estimate the parameters in the CBRM:

### Algorithm 4.1 (penalty-based coordinate ascent algorithm for CBRM):

*Initialization*: set $\delta_{0}=1$ , starting point to ${\hat{\Theta }}_{0}$ $\overset{yields}{\to }$ $\varphi ({\hat{\Theta }}_{0},1)$ , and ε→0 ;

**for** k=1,2,…

 *Maximization*: Find the coordinate ascent maximizer ${\hat{\Theta }}_{k}$ of $\varphi (\Theta ,\delta_{k})$ starting at ${\hat{\Theta }}_{k-1}$ such that

  **for** i=1,2,…

    Using the NR algorithm, to find

${\hat{\rho }}_{i}=\underset{\rho }{\arg\;\max}\;\varphi (\rho ;{\hat{\alpha }}_{i-1},\;{\hat{\beta }}_{i-1},\;\hat{\sigma }{{}_{a}^{2}}_{i-1},\hat{\sigma }{{}_{b}^{2}}_{i-1},\delta_{k})$ ,

$\hat{\sigma }a_{i}^{2}=\underset{\sigma_{a}^{2}}{\arg\;\max}\;\varphi (\sigma_{a}^{2};{\hat{\alpha }}_{i-1},\;{\hat{\beta }}_{i-1},\;\hat{\sigma }{{}_{b}^{2}}_{i-1},{\hat{\rho }}_{i},\delta_{k}),$

$\hat{\sigma }b_{i}^{2}=\underset{\sigma_{b}^{2}}{\arg\;\max}\;\varphi (\sigma_{b}^{2};\;{\hat{\alpha }}_{i-1},\;{\hat{\beta }}_{i-1},\;\hat{\sigma }{{}_{a}^{2}}_{i},{\hat{\rho }}_{i},\delta_{k})$ ,

${\hat{\alpha }}_{i}=\underset{\alpha }{\arg\;\max}\;\varphi (\alpha ;{\hat{\beta }}_{i-1},\hat{\sigma }{{}_{a}^{2}}_{i},\hat{\sigma }{{}_{b}^{2}}_{i},{\hat{\rho }}_{i},\delta_{k}),$

${\hat{\beta }}_{i}=\underset{\beta }{\arg\;\max}\;\varphi (\beta ;\;{\hat{\alpha }}_{i},\;\hat{\sigma }{{}_{a}^{2}}_{i},\hat{\sigma }{{}_{b}^{2}}_{i},{\hat{\rho }}_{i},\delta_{k}),$

    and ${\hat{\Theta }}_{i}=\;({\hat{\alpha }}_{i},\;{\hat{\beta }}_{i},\;\hat{\sigma }{{}_{a}^{2}}_{i},\hat{\sigma }{{}_{b}^{2}}_{i},{\hat{\rho }}_{i}{)}^{T}$

    *Convergence*: Terminating when $|{\hat{\Theta }}_{i}-{\hat{\Theta }}_{i-1}|<\varepsilon$ ;

    **if** final convergence test satisfied

      **stop** with approximate solution ${\hat{\Theta }}_{i}\;=\;({\hat{\alpha }}_{i},\;{\hat{\beta }}_{i},\;\hat{\sigma }{{}_{a}^{2}}_{i},\hat{\sigma }{{}_{b}^{2}}_{i},{\hat{\rho }}_{i}{)}^{T}$ ;

    **end** (**if**)

  **end** (**for**)

  set ${\hat{\Theta }}_{k}={\hat{\Theta }}_{i}$

  *Penalty Convergence*: Terminating when $|{\hat{\Theta }}_{k}-{\hat{\Theta }}_{k-1}|\leq \varepsilon$ ;

  **if** final convergence test satisfied

    **stop** with approximate solution ${\hat{\Theta }}_{k}$ ;

  **end** (**if**)

  *Updating*: Set new penalty parameter $\delta_{k+1}$ = 10 $\delta_{k}$ ;

        Set new starting point to ${\hat{\Theta }}_{k}$ ;

**end** (**for**)

Although the algorithm can be computationally expensive, this may be controlled by applying large step changes to the penalty coefficient $\delta_{k}$ thereby ensuring the parameter estimates are largely contained in the feasible region. Occasionally when the data are noisy, unfeasible values such as an infinite derivative or negative variance may emerge during the iterative process. Normally, this may be remedied by resetting the responsible parameter to either the initial value or to the value in a previous iteration. This allows the next iteration to proceed on a slightly modified trajectory, which enhances the algorithm convergence.

## 5 Numerical examples

To evaluate the algorithm for the CBRM, we present a simulation study that compares it with another NR based algorithm for the BRM.^3^ The performance characteristics are measured in terms of mean bias, mean squared error, coverage probability, and convergence probability. The ‘closeness’ of the estimates for the sensitivity and specificity to their true values are measured using the Euclidian and Mahalanobis distances. In addition, we apply the algorithm to two real data examples. The codes and analyses were conducted in R.^17^

### 5.1 Simulated data examples

A simulation study was conducted to compare the CBRM with the BRM. For the simulated data, the parameters α and β were obtained by taking the logits of *s* and 1−f respectively, where *s* and *f* were simulated from a uniform distribution on the interval [0, 1] so that they satisfied the condition, f<s .

For the ith study (i=1,…,k), to simulate $\alpha_{i}$ and $\beta_{i}$ we assumed the bivariate normal distribution in (12) such that:

(8) $$\left(\begin{array}{c} \alpha_{i}\\ \beta_{i} \end{array}\right)∼N\left(\left(\begin{array}{c} \alpha {|}_{\;f<\;s}\\ \beta {|}_{\;f<\;s} \end{array}\right),\;\left(\begin{array}{cc} \tau^{2} & \rho \tau^{2}\\ \rho \tau^{2} & \tau^{2} \end{array}\right)\right)$$

where $\tau^{2}=\sigma_{a}^{2}=\sigma_{b}^{2}$ . The number of non-diseased $n_{b,i}$ , were randomly sampled from uniform distribution with equal probability and with replacement from the set of integers between 50 and 1000 inclusive. The number of diseased $n_{a,i}$ , were chosen to be $\gamma .n_{b,i}$ rounded to the nearest whole number, where γ was randomly simulated to be between 0.05 and 0.5. The true positives $TP_{i}$ , and true negatives $TN_{i}$ were simulated from the binomial distributions detailed in equations (2) and (3).

For the clinical setting of interest, one more study with overall sample size *n* was simulated to provide $\alpha_{k+1}$ , $\beta_{k+1}$ , $n_{b,k+1}$ , $TP_{k+1}$ , and $TN_{k+1}$ . Here $n_{a,k+1}$ was obtained from $n-n_{b,k+1}$ . From these the upper and lower 99% confidence limits for *r*, $\left[r_{l},\;r_{u}\right]$ and *p*, $\left[p_{l},p_{u}\right]$ respectively, were estimated using methods described elsewhere.^18^ These were used to define the constrained region for the setting of interest and the constrained model.

The simulation study was conducted in two stages. For the first stage, the four parameters $\left\{k,\tau^{2},\rho ,n\right\}$ were set to {10, 0.5, −0.5, 100} respectively to represent a moderate sized meta-analysis with moderate heterogeneity, moderate correlation between the sensitivity and specificity and a moderate amount of information from the setting. For each scenario, a single parameter was then varied whilst the others remained fixed at these values. Thus, the relevant parameters were fixed to the following: the number of studies, k={5,10,20,50} ; to capture different levels of heterogeneity, with 0.1 indicating low heterogeneity and 1.5 extensive heterogeneity, the variances $\tau^{2}=\{0.1,0.5,1.0,1.5\}$ ; the correlation between the logits of the sensitivity and specificity, ρ={−0.1,−0.25,−0.5,−0.75,−0.9} ; and to reflect different levels of information from the setting of interest, the setting-specific sample size, n={50,100,250,500,1000} .

For the second stage, we considered more extreme scenarios than in the first stage. In this stage, each scenario was a combination of the minimum or maximum of each parameter's values of the previous simulation. Thus we considered all 16 possible combinations between *n* = {50, 10000}, *k* = {5, 50}, $\tau^{2}=\{0.1,1.5\}$ , and ρ={−0.1,−0.90} . For all scenarios, 10,000 simulated samples datasets were generated.

Table 1 provides the mean bias and the mean squared errors (MSE) for the estimated values of sensitivity and specificity for each scenario in the first stage. In all but one of the scenarios, the CBRM returns estimates for the sensitivity that have a lower absolute mean bias than the BRM. Similarly for the specificity, the CBRM returns estimates that have a lower absolute mean bias than the BRM in all but three of the scenarios. In general, for the MSE, the CBRM returns estimates that have a lower MSE than the BRM across all scenarios.

**Table 1.** Mean bias and mean squared error of the estimated values of sensitivity and specificity for the CBRM and BRM based on10000 simulations for each of the scenarios.

		Bias				Mean Squared Error			
		Sensitivity		Specificity		Sensitivity		Specificity	
		CBRM	BRM	CBRM	BRM	CBRM	BRM	CBRM	BRM
* *	{ *k*, *τ^2^, ρ* }								
*n* = 50	{10, 0.5, −0.5}	−0.0097	−0.0112	−0.0016	−0.0022	0.0171	0.0182	0.0130	0.0141
*n* = 100	{10, 0.5, − 0.5}	−0.0075	−0.0100	−0.0026	−0.0033	0.0152	0.0177	0.0104	0.0134
*n* = 250	{10, 0.5, − 0.5}	−0.0068	−0.0093	−0.0037	−0.0043	0.0124	0.0175	0.0070	0.0133
*n* = 500	{10, 0.5, − 0.5}	−0.0074	−0.0101	−0.0031	−0.0035	0.0101	0.0177	0.0050	0.0134
*n* = 1000	{10, 0.5, − 0.5}	−0.0098	−0.0105	−0.0052	−0.0057	0.0086	0.0182	0.0035	0.0137
* *	{ *τ^2^, ρ, n* }								
*k* = 5	{0.5, − 0.5, 100}	−0.0086	−0.0119	−0.0033	−0.0048	0.0171	0.0204	0.0112	0.0151
*k* = 10	{0.5, − 0.5, 100}	−0.0075	−0.0100	−0.0026	−0.0033	0.0152	0.0177	0.0104	0.0134
*k* = 20	{0.5, − 0.5, 100}	−0.0100	−0.0115	−0.0025	−0.0035	0.0145	0.0166	0.0104	0.0132
*k* = 50	{0.5, − 0.5, 100}	−0.0084	−0.0103	−0.0025	−0.0027	0.0139	0.0160	0.0101	0.0128
	{ *k*, *ρ, n* }								
$\tau^{2}=$ 0.1	{10,− 0.5, 100}	−0.0022	−0.0025	−0.0001	−0.0003	0.0039	0.0040	0.0029	0.0030
$\tau^{2}=$ 0.5	{10,− 0.5, 100}	−0.0075	−0.0100	−0.0026	−0.0033	0.0152	0.0177	0.0104	0.0134
$\tau^{2}=$ 1.0	{10,− 0.5, 100}	−0.0167	−0.0223	−0.0032	−0.0031	0.0248	0.0327	0.0148	0.0236
$\tau^{2}=$ 1.5	{10,− 0.5, 100}	−0.0178	−0.0275	−0.0047	−0.0039	0.0330	0.0470	0.0186	0.0345
	{ *k*, *τ^2^, n* }								
ρ = −0.10	{10, 0.5, 100}	−0.0180	−0.0204	−0.0090	−0.0108	0.0156	0.0169	0.0102	0.0122
ρ = −0.25	{10, 0.5, 100}	−0.0150	−0.0175	−0.0071	−0.0083	0.0156	0.0173	0.0106	0.0130
ρ = −0.50	{10, 0.5, 100}	−0.0075	−0.0100	−0.0026	−0.0033	0.0152	0.0177	0.0104	0.0134
ρ = −0.75	{10, 0.5, 100}	0.0002	−0.0019	0.0011	0.0006	0.0145	0.0179	0.0102	0.0139
ρ = −0.90	{10, 0.5, 100}	0.0026	−0.0002	0.0049	0.0059	0.0142	0.0186	0.0101	0.0145

As expected, the differences in performance between the models in terms of the MSE are most pronounced for large *n* – as *n* increases the constrained region becomes more precise thus lowering the MSE for the CBRM.

From the same table we can see that, for both the CBRM and BRM, increasing the value of the variances $\tau^{2}$ in the simulations lead to increasing the values of bias and MSE for the sensitivity and specificity. Also when we increase the correlation level ρ , we get higher values of the bias for sensitivity and specificity.

The mean bias and MSE for the between-study covariance matrix parameters are given in Table A1 and A2 of the Appendix. Table A1 gives the mean bias for the estimated values of ${\hat{\sigma }}_{a}^{2}$ , ${\hat{\sigma }}_{b}^{2}$ and $\hat{\rho }$ . In all but three of the different scenarios, the CBRM returns estimated values of ${\hat{\sigma }}_{a}^{2}$ and ${\hat{\sigma }}_{b}^{2}$ with less absolute bias compared to the BRM. However, the MSEs of ${\hat{\sigma }}_{a}^{2}$ and ${\hat{\sigma }}_{b}^{2}$ , in general, are larger for the CBRM compared with the BRM. Furthermore it is also noted that the BRM underestimates the variance across all scenarios, whilst the CBRM underestimates the variance across all but 3 scenarios.

The prediction regions provide the region where we would expect to find the parameters $\left(\alpha_{k+1},\;\beta_{k+1}\right)$ for a new study. As the constrained model uses setting-specific information, it may be expected to provide better coverage than the BRM. Table 2 gives the coverage probability of the 95% confidence and prediction regions and the convergence probability for each scenario in the first stage.

**Table 2.** The coverage probability of the 95% confidence and prediction regions and the convergence probability for each scenario for the CBRM and BRM based on 10000 simulations.

		Confidence Regions		Prediction Regions		Convergence Probability	
		CBRM	BRM	CBRM	BRM	CBRM	BRM
* *	{ *k*, *τ^2^, ρ* }						
*n* = 50	{10, 0.5, − 0.5}	0.3393	0.3313	0.9035	0.8964	0.9992	0.9999
*n* = 100	{10, 0.5, − 0.5}	0.3531	0.3339	0.9206	0.9023	0.9982	1.0000
*n* = 250	{10, 0.5, − 0.5}	0.4031	0.3227	0.9288	0.9012	0.9954	1.0000
*n* = 500	{10, 0.5, − 0.5}	0.4765	0.3351	0.9390	0.9004	0.9896	1.0000
*n* = 1000	{10, 0.5, − 0.5}	0.5402	0.3350	0.9431	0.9005	0.9877	1.0000
* *	{ *τ^2^, ρ, n* }						
*k* = 5	{0.5, − 0.5, 100}	0.6869	0.6449	0.9231	0.9109	0.9958	1.0000
*k* = 10	{0.5, − 0.5, 100}	0.3531	0.3339	0.9206	0.9023	0.9982	1.0000
*k* = 20	{0.5, − 0.5, 100}	0.1876	0.1820	0.9395	0.9225	0.9989	1.0000
*k* = 50	{0.5, − 0.5, 100}	0.0831	0.0816	0.9563	0.9416	0.9979	1.0000
	{ *k*, *ρ, n* }						
$\tau^{2}=$ 0.1	{10,− 0.5, 100}	0.4105	0.4071	0.8797	0.8774	0.9992	1.0000
$\tau^{2}=$ 0.5	{10,− 0.5, 100}	0.3531	0.3339	0.9206	0.9023	0.9982	1.0000
$\tau^{2}=$ 1.0	{10,− 0.5, 100}	0.3686	0.3191	0.9317	0.9066	0.9950	1.0000
$\tau^{2}=$ 1.5	{10,− 0.5, 100}	0.3816	0.3102	0.9344	0.9057	0.9911	1.0000
	{ *k*, *τ^2^, n* }						
ρ = −0.10	{10, 0.5, 100}	0.3472	0.3320	0.9121	0.8981	0.9980	1.0000
ρ = −0.25	{10, 0.5, 100}	0.3447	0.3275	0.9150	0.8981	0.9970	1.0000
ρ = −0.50	{10, 0.5, 100}	0.3531	0.3339	0.9206	0.9023	0.9982	1.0000
ρ = −0.75	{10, 0.5, 100}	0.3691	0.3477	0.9230	0.9049	0.9984	1.0000
ρ = −0.90	{10, 0.5, 100}	0.4029	0.3750	0.9213	0.8986	0.9970	0.9999

Clearly, for all scenarios, the CBRM returns higher coverage probabilities of the 95% confidence and prediction regions for $\left(\alpha_{k+1},\beta_{k+1}\right)$ compared to the BRM. As the setting-specific sample size *n*, increases the coverage probabilities of the 95% confidence and prediction regions for CBRM also increase. On the other hand, as the distribution of studies becomes more dispersed (by increasing $\tau^{2}$ ) the coverage probabilities of the confidence region in the BRM decreases whilst the coverage of the prediction region increases.

The BRM model is considered to have converged when the algorithm settles on a stable estimate before reaching a specific number of iterations. For the CBRM model to have converged, the sensitivity and specificity estimates must also satisfy the conditions in (5) and hence be located in the applicable region. The effect of this is seen in the last two columns of table 2 which show the probability of convergence for the CBRM to be slightly lower than the BRM.

As a measure of the deviation, we calculated the distance between the true values for the false positive rate and sensitivity $\left(f_{k+1},s_{k+1}\right)$ , for a new study and its summary estimated pair $\left(\hat{\;f},\hat{s}\right)$ from each model, where $f_{k+1}=1-logit^{-1}(\beta_{k+1})$ , $s_{k+1}=logit^{-1}(\alpha_{k+1})$ , $\hat{\;f}=1-logit^{-1}(\hat{\beta })$ , and $\hat{s}=logit^{-1}(\hat{\alpha })$ . For this purpose, both the Euclidean distance and Mahalanobis distance were used as metrics.^19^

The distributions for the Euclidean distances are shown in figures A1, A2, A3 and A4 in the Appendix. It is clear for the CBRM that the lower quartile limit, median, upper quartile limit, 99.5 percentile and maximum are lower than the BRM. Whilst for some of the scenarios the difference between the CBRM and BRM is subtle, as the setting-specific sample size, *n* increases the difference in the medians in particular becomes more marked.

As a head to head comparison we considered which model was most likely to provide an estimate for $\left(\hat{\;f}.\hat{s}\right)$ which was closest to the true estimate $\left(f_{k+1}.s_{k+1}\right)$ in terms of the Euclidean and Mahalanobis distances.^19^ Where distances were the same between the two models we considered the model estimates to be equivalent. The results are in table 3 and clearly demonstrate that the CBRM has the highest probabilities of shortest distance for all scenarios. Furthermore as the constrained region narrows and becomes more precise the CBRM is more likely to provide an estimate which is closest to the true value than the BRM.

**Table 3.** The probabilities of shortest distance between the estimated $\left(\hat{\;f},\hat{s}\right)$ and the true value $\left(f_{k+1},s_{k+1}\right)$ , for the BRM and CBRM based on the euclidian and mahalanobis distances. “Equivalent” is where the estimates from the two models were the same to 12 decimal places for each of the scenarios.

		Euclidean distance			Mahalanobis distance		
		Equivalent	BRM	CBRM	Equivalent	BRM	CBRM
	{ *k*, *τ^2^, ρ* }						
*n* = 50	{10, 0.5, − 0.5}	0.0241	0.4633	0.5126	0.0231	0.4609	0.5160
*n* = 100	{10, 0.5, − 0.5}	0.0194	0.4229	0.5577	0.0189	0.4204	0.5607
*n* = 250	{10, 0.5, − 0.5}	0.0164	0.3380	0.6456	0.0162	0.3419	0.6419
*n* = 500	{10, 0.5, − 0.5}	0.0178	0.2644	0.7178	0.0177	0.2755	0.7068
*n* = 1000	{10, 0.5, − 0.5}	0.0125	0.2132	0.7743	0.0120	0.2245	0.7635
	{ *τ^2^, ρ, n* }						
*k* = 5	{0.5, − 0.5, 100}	0.0211	0.4065	0.5724	0.0207	0.4220	0.5573
*k* = 10	{0.5, − 0.5, 100}	0.0194	0.4229	0.5577	0.0189	0.4204	0.5607
*k* = 20	{0.5, − 0.5, 100}	0.0192	0.4242	0.5566	0.0189	0.4226	0.5585
*k* = 50	{0.5, − 0.5, 100}	0.0115	0.4319	0.5566	0.0109	0.4300	0.5591
	{ *k*, *ρ, n* }						
$\tau^{2}=$ 0.1	{10,− 0.5, 100}	0.0241	0.4788	0.4971	0.0230	0.4729	0.5041
$\tau^{2}=$ 0.5	{10,− 0.5, 100}	0.0194	0.4229	0.5577	0.0189	0.4204	0.5607
$\tau^{2}=$ 1.0	{10,− 0.5, 100}	0.0171	0.3658	0.6171	0.0168	0.3662	0.6170
$\tau^{2}=$ 1.5	{10,− 0.5, 100}	0.0133	0.3348	0.6519	0.0132	0.3393	0.6475
	{ *k*, *τ^2^, n* }						
ρ = −0.10	{10, 0.5, 100}	0.0406	0.4132	0.5462	0.0400	0.4196	0.5404
ρ = −0.25	{10, 0.5, 100}	0.0356	0.4170	0.5474	0.0349	0.4213	0.5438
ρ = -0.50	{10, 0.5, 100}	0.0194	0.4229	0.5577	0.0189	0.4204	0.5607
ρ = −0.75	{10, 0.5, 100}	0.0116	0.4118	0.5766	0.0113	0.4077	0.5810
ρ = −0.90	{10, 0.5, 100}	0.0173	0.4194	0.5633	0.0166	0.4138	0.5696

For the second stage of the simulations, where we considered more extreme scenarios, the mean bias and the mean squared errors (MSE) for the estimated values of sensitivity and specificity are given in table 4. In all but three of the scenarios, the CBRM returns estimates for the specificity that have a lower absolute mean bias than the BRM. For the sensitivity, the CBRM returns estimates that have a lower absolute mean bias than the BRM in eight of the scenarios. In addition, for the MSE, the CBRM returns estimates that have a lower MSE than the BRM across all scenarios except four where they were equal. As with the stage 1 simulations, the most influential parameter is *n*, the sample size of the routine data collected from the setting of interest Thus, when *n* = 1000, the CBRM returns estimates for both sensitivity and specificity that have a lower MSE than the BRM over all the scenarios.

**Table 4.** Mean bias and mean squared error of the estimated values of sensitivity and specificity for the CBRM and BRM based on10000 simulations for each of the 16 scenarios in stage 2.

				Mean Bias				Mean Squared Error			
				Sensitivity		Specificity		Sensitivity		Specificity	
*n*	k	$\tau^{2}$	ρ	CBRM	BRM	CBRM	BRM	CBRM	BRM	CBRM	BRM
50	5	0.1	−0.1	−0.0037	−0.0044	−0.0025	−0.0029	0.0045	0.0046	0.0032	0.0032
50	5	0.1	−0.9	0.0018	0.0015	−0.0000	−0.0001	0.0047	0.0047	0.0035	0.0035
50	5	1.5	−0.1	−0.0380	−0.0476	−0.0207	−0.0268	0.0429	0.0488	0.0277	0.0353
50	5	1.5	−0.9	0.0061	−0.0026	0.0103	0.0118	0.0380	0.0512	0.0281	0.0413
50	50	0.1	−0.1	−0.0046	−0.0047	−0.0017	−0.0017	0.0035	0.0035	0.0027	0.0027
50	50	0.1	−0.9	0.0004	0.0003	0.0015	0.0015	0.0037	0.0037	0.0029	0.0029
50	50	1.5	−0.1	−0.0384	−0.0418	−0.0222	−0.0235	0.0372	0.0399	0.0245	0.0282
50	50	1.5	−0.9	0.0021	−0.0042	0.0175	0.0215	0.0328	0.0423	0.0253	0.0349
1000	5	0.1	−0.1	−0.0028	−0.0041	−0.0021	−0.0031	0.0039	0.0047	0.0020	0.0033
1000	5	0.1	−0.9	0.0017	0.0011	0.0004	0.0004	0.0026	0.0046	0.0016	0.0035
1000	5	1.5	−0.1	−0.0396	−0.0456	−0.0211	−0.0305	0.0264	0.0493	0.0083	0.0360
1000	5	1.5	−0.9	−0.0020	−0.0015	0.0003	0.0107	0.0088	0.0502	0.0034	0.0407
1000	50	0.1	−0.1	−0.0043	−0.0046	−0.0029	−0.0034	0.0030	0.0035	0.0017	0.0026
1000	50	0.1	−0.9	0.0008	0.0001	0.0010	0.0015	0.0020	0.0035	0.0014	0.0027
1000	50	1.5	−0.1	−0.0423	−0.0408	−0.0238	−0.0271	0.0213	0.0390	0.0069	0.0275
1000	50	1.5	−0.9	−0.0022	−0.0003	0.0005	0.0157	0.0068	0.0412	0.0031	0.0339

Table A3 and A4 gives the mean bias and MSE for the estimated values of ${\hat{\sigma }}_{a}^{2}$ , ${\hat{\sigma }}_{b}^{2}$ and $\hat{\rho }$ . In half of the scenarios, the CBRM returns estimated values of ${\hat{\sigma }}_{a}^{2}$ and ${\hat{\sigma }}_{b}^{2}$ with less absolute bias compared to the BRM. In contrast, the MSEs of ${\hat{\sigma }}_{a}^{2}$ and ${\hat{\sigma }}_{b}^{2}$ are larger for the CBRM compared with the BRM in 14 of the 16 scenarios.

Table 5 gives the coverage probability of the 95% confidence and prediction regions and the convergence probability for the corresponding simulations. Again, for all scenarios, the CBRM returns higher coverage probabilities of the 95% confidence and prediction regions for $\left(\alpha_{k+1},\beta_{k+1}\right)$ compared to the BRM.

**Table 5.** The coverage probability of the 95% confidence and prediction regions and the convergence probability for each of the 16 scenario for the CBRM and BRM based on10000 simulations.

				Confidence Regions		Prediction Regions		Convergence Probability	
n	k	$\tau^{2}$	ρ	CBRM	BRM	CBRM	BRM	CBRM	BRM
50	5	0.1	−0.1	0.7503	0.7470	0.9305	0.9301	0.9992	1.0000
50	5	0.1	−0.9	0.8050	0.8030	0.9526	0.9514	0.9988	0.9998
50	5	1.5	−0.1	0.6341	0.5998	0.9198	0.9083	0.9913	1.0000
50	5	1.5	−0.9	0.6983	0.6419	0.9310	0.9069	0.9909	1.0000
50	50	0.1	−0.1	0.1018	0.1013	0.9172	0.9170	0.9998	1.0000
50	50	0.1	−0.9	0.1379	0.1369	0.8958	0.8921	0.9978	1.0000
50	50	1.5	−0.1	0.0759	0.0749	0.9461	0.9287	0.9958	1.0000
50	50	1.5	−0.9	0.0813	0.0805	0.9482	0.9351	0.9950	1.0000
									
1000	5	0.1	−0.1	0.8201	0.7485	0.9541	0.9402	0.9938	0.9999
1000	5	0.1	−0.9	0.9071	0.8135	0.9787	0.9571	0.9776	0.9999
1000	5	1.5	−0.1	0.7741	0.5901	0.9478	0.9042	0.9665	0.9999
1000	5	1.5	−0.9	0.8671	0.6554	0.9740	0.9244	0.9677	1.0000
1000	50	0.1	−0.1	0.0996	0.0946	0.9433	0.9189	0.9949	1.0000
1000	50	0.1	−0.9	0.1543	0.1451	0.9348	0.9027	0.9646	1.0000
1000	50	1.5	−0.1	0.1800	0.0778	0.9700	0.9323	0.9688	1.0000
1000	50	1.5	−0.9	0.2349	0.0865	0.9721	0.9371	0.9735	1.0000

From table 6, it can be clearly seen that the CBRM has the highest probabilities of shortest distance for all scenarios using the Mahalanobis distance metric and for all except one scenario using the Euclidean distance metric. Furthermore, the distributions for the Euclidean distances that are shown in figures A5, A6, A7 and A8 in the Appendix, illustrate that the CBRM's lower quartile limit, median, upper quartile limit, 99.5 percentile and maximum are lower than the BRM.

**Table 6.** The probabilities of shortest distance between the estimated $\left(\hat{f},\hat{s}\right)$ and the true estimate $\left(f_{k+1},s_{k+1}\right)$ , for the BRM and CBRM based on the euclidian and mahalanobis distances. “Equivalent” is where the estimates from the two models were the same to 12 decimal places for each of the 16 scenarios.

				Euclidean distance			Mahalanobis distance		
n	k	$\tau^{2}$	ρ	Equivalent	BRM	CBRM	Equivalent	BRM	CBRM
50	5	0.1	−0.1	0.0268	0.4807	0.4925	0.0257	0.4871	0.4872
50	5	0.1	−0.9	0.0777	0.4655	0.4568	0.0765	0.4571	0.4664
50	5	1.5	−0.1	0.0192	0.3946	0.5862	0.0189	0.4036	0.5775
50	5	1.5	−0.9	0.0189	0.3944	0.5867	0.0189	0.4205	0.5606
50	50	0.1	−0.1	0.0925	0.4520	0.4555	0.0906	0.4507	0.4587
50	50	0.1	−0.9	0.0368	0.4819	0.4813	0.032	0.4809	0.4871
50	50	1.5	−0.1	0.0410	0.4161	0.5429	0.0406	0.4143	0.5451
50	50	1.5	−0.9	0.0077	0.3964	0.5959	0.0069	0.3996	0.5935
									
1000	5	0.1	−0.1	0.0229	0.3630	0.6141	0.0223	0.3724	0.6053
1000	5	0.1	−0.9	0.0642	0.3059	0.6299	0.0635	0.3320	0.6045
1000	5	1.5	−0.1	0.0061	0.1805	0.8134	0.0061	0.2045	0.7894
1000	5	1.5	−0.9	0.0067	0.1284	0.8649	0.0067	0.2125	0.7808
1000	50	0.1	−0.1	0.0658	0.3592	0.5750	0.0645	0.3602	0.5753
1000	50	0.1	−0.9	0.0340	0.3491	0.6169	0.0309	0.3539	0.6152
1000	50	1.5	−0.1	0.0124	0.1637	0.8239	0.0124	0.1585	0.8291
1000	50	1.5	−0.9	0.0040	0.1346	0.8614	0.0038	0.1694	0.8268

### 5.2 Real data examples

Here, the CBRM algorithm is applied to two real meta-analysis datasets.^2,20,21^ In each meta-analysis, the CBRM parameters were estimated and compared with estimates for the parameters in the BRM.

The first meta-analysis evaluates the performance of computed tomography (CT) in detecting distant metastases.^20^ There are 12 studies and the focus here will be on estimating the parameters in the CBRM using the penalty-based co-ordinate ascent algorithm and defining the applicable region for the 12 studies. The lower and upper limits of *r* are taken to be 0.24 and 0.4 respectively, while the lower and upper limits of *p* are taken to be 0.01 and 0.05 respectively.

The second meta-analysis evaluates the performance of Centor's criteria for diagnosing streptococcal infection in patients presenting to primary care with a sore throat.^21^ There were nine studies included in the analysis. The upper and lower limits of *r* and *p* are based on data collected from a general practice setting and define an applicable region of feasible sensitivities and specificities for the setting in question.^2^

**Figure 1.**
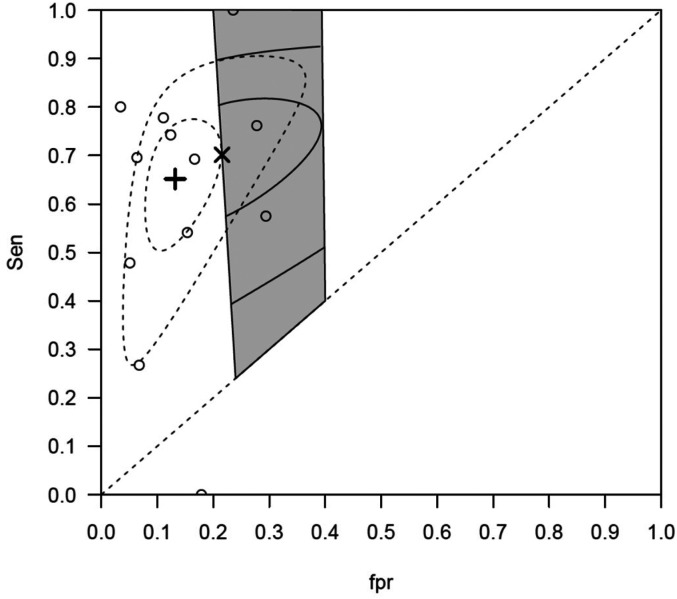
CT data: The studies are presented in circles ○. The grey shaded area represents the applicable region for the general practice. The summary estimate of BRM is represented by the **+ **, its confidence region is the inner dashed ellipse and prediction region is the outer dashed ellipse. The summary estimate of CBRM is represented by the x. Its confidence region is the inner continuous ellipse truncated by the left boundary of the applicable region. Its prediction region is the outer continuous ellipse truncated by the left and right boundaries of the applicable region.

**Figure 2.**
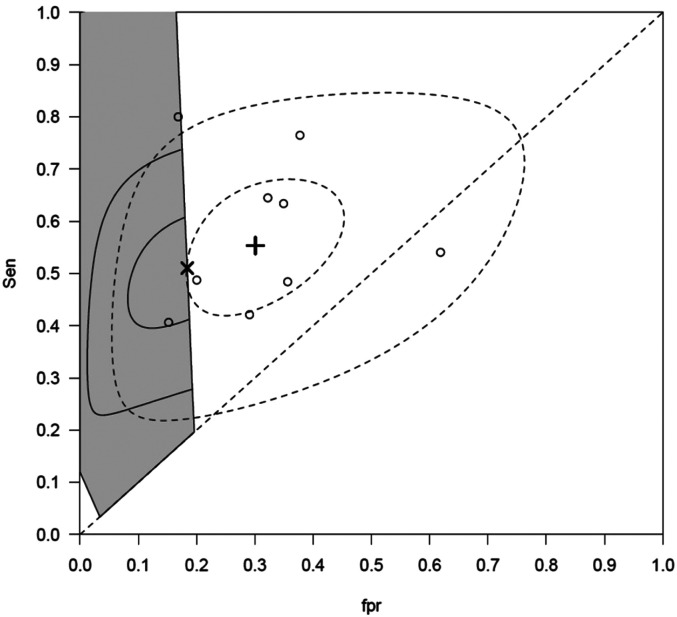
Centor data: The studies are presented in circles ○. The grey shaded area represents the applicable region for the general practice. The summary estimate of the BRM is represented by the **+ **, its confidence region is the inner dashed ellipse and prediction region is the outer dashed ellipse. The summary estimate of CBRM is represented by the x. Its confidence region is the inner continuous ellipse truncated by the right boundary of the applicable region, and its prediction region is the outer continuous ellipse truncated by the right boundary of the applicable region.

Figures 1 and 2 illustrate the bivariate plots for the first and second meta-analysis respectively. In each case, it can be seen that the point estimate for the BRM lies outside of the constrained region. Since there is a less than 1% chance that the space outside the constrained region contains the true sensitivity and specificity for the test in the setting, the BRM estimates in these two cases are unlikely to be representative for the settings of interest

In contrast, the estimates for the CBRM lie on the boundaries of the respective constrained regions and therefore are strictly within the region and so are more likely to represent the setting of interest. Furthermore, in Figure 2 there are only two studies in the constrained region and it is likely there would have been too few studies to aggregate using a standard tailored meta-analysis approach. In table 7, the estimates for the five parameters for the CBRM algorithm and the BRM algorithm are given. It is clear the two models give different estimates for the two datasets.

**Table 7.** Estimates for α , β $\sigma_{a}^{2},\sigma_{b}^{2}$ and ρ (in logit space) for the CBRM and BRM models for the CT and Centor datasets.

Data				Estimate	
	Model	Sensitivity	Specificity	${\hat{\sigma }}_{a}^{2}$	${\hat{\sigma }}_{b}^{2}$	$\hat{\rho }$
CT	CBRM	0.7015	0.7843	0.3274	0.5945	−0.6800
CT	BRM	0.6515	0.8678	0.2794	0.1736	−0.7743
						
Centor	CBRM	0.5104	0.8161	0.1440	0.7860	−0.6108
Centor	BRM	0.5535	0.6993	0.2037	0.3815	−0.4771

## 6 Discussion

Although meta-analysis, in general, has proved a successful tool in informing the evidence base on interventions, its role in summarising the performance of a test is more nuanced. For instance, the question of “how accurate is this test when applied to patients in our hospital?” is unlikely to be answered by a test accuracy meta-analysis. The presence of heterogeneity, a common feature in test accuracy meta-analyses,^11^ ensures that in many cases the summary estimate is not representative of an individual clinical setting.

Ultimately this affects the extent in which we may use secondary research to assist diagnosis.

In order to improve the utility of meta-analysis in diagnostic prediction requires modifying the current approach. To this end tailored meta-analysis adapts conventional meta-analysis by incorporating evidence from the setting of interest to make it more relevant clinical practice.^2^

Thus, the main objective of tailored meta-analysis is to find a plausible estimate for a diagnostic test's performance through defining an applicable region based on data collected from the clinical setting.^2^ Specifically, an estimate for the local test positive rate and local prevalence allows a plausible estimate for the test performance in the setting of interest to be derived from the meta-analysis. Potentially this could improve diagnostic prediction.

In previous analyses the constrained ‘applicable region’ has been used to select only those studies that are deemed relevant for the setting.^1,2,18^ leading to some of the studies to be excluded from the meta-analysis. As a result this may lead to a loss of information. In contrast, the constrained model proposed here, includes all of the studies. The resulting model presents a challenging nonlinear constrained optimization problem which, like the BRM, has no closed form for the likelihood function.

In order to derive the maximum likelihood estimate, we have developed a penalty-based algorithm for estimating the parameters in the CBRM. It starts with analytical estimates of the initial values for a Newton-Raphson based coordinate ascent algorithm used to maximize the penalty function. The algorithm continues by updating the penalty parameter and the starting point until convergence is achieved.

Based on the simulation study and the two previously published test accuracy reviews, the algorithm is capable of providing plausible estimates for the parameters in the constrained bivariate random effects model. This contrasts the unconstrained approach where for the two real examples the summary estimates for the sensitivity and specificity were both outside the constrained region.

Measuring the validity of estimates produced by meta-analysis models is an area of active research where the methods usually incorporate ‘leave-one-out’ or leave-k-out’ cross-validation techniques.^22^ Although the statistical basis for these is well developed, they tend to apply to univariate models.^22^ Here, through simulation studies, we used the Euclidean and Mahalanobis distance metrics to measure the ‘closeness’ of the model estimates for the sensitivity and specificity with their true values. These studies demonstrated that compared with the BRM, the CBRM is more likely to provide a point estimate for the sensitivity and specificity which is closer to the true values for a new study setting. They also showed that the CBRM is more likely to estimate a prediction region to capture the true sensitivity and specificity for a new study.

However, the model is not without limitations. Previous research has demonstrated the effects of outlier studies on the BRM and proposed methods for dealing with outliers and providing robust estimates for the model's main parameters.^23,24^ Although not investigated here, outlier studies could also affect the estimates of the CBRM and future research could investigate incorporating methods for dealing with outliers in the CBRM.

Both models are essentially generalized mixed models and a Maximum Likelihood (ML) approach to estimating the parameters was implemented. Other approaches such as the Restricted Maximum Likelihood (REML) have been used in linear mixed models as they provide bias-corrected estimates for the variances. However, REML approaches to generalized linear models are less well developed and some packages such as lme4 do not implement them on this basis.^6^ Nonetheless, it is clear that a ML approach to the BRM (and CBRM) underestimates the variance suggesting research into other approaches is needed for these models.

The penalty method is not the only approach that could be used to optimize the CBRM. Other numerical nonlinear optimization algorithms such as the barrier method or augmented Lagrangian method could be used^10^ and like the penalty method, these need to be evaluated for the constrained model over a range of simulated examples. Potentially, any improvements on the computation time or mean-squared error may give the methods priority over the penalty method.

Furthermore, other NR based approaches may be used such as the expected Fisher Information matrix,^25^ which provides a positive definite Hessian matrix. This has the advantage of generating an estimate for each of the five parameters simultaneously at each iteration and could potentially save on computational time.

Although the benefits of using specific quantitative information from clinical practice to augment meta-analysis have already been demonstrated the methods used so far have focussed on modifying study selection. Here we have proposed a constrained model that incorporates all the studies and guarantees that the summary estimate for the sensitivity and specificity is located in a plausible or applicable region for the practice setting. As such, it has the potential to improve diagnostic decision-making in practice.

## Supplemental Material

sj-docx-1-smm-10.1177_09622802211065157 - Supplemental material for On estimating a constrained bivariate random effects model for meta-analysis of test accuracy studies Supplemental material, sj-docx-1-smm-10.1177_09622802211065157 for On estimating a constrained bivariate random effects model for meta-analysis of test accuracy studies by Mohammed Baragilly and Brian Harvey Willis in Statistical Methods in Medical Research
