# Supplementary material for: On estimating a constrained bivariate random effects model for meta-analysis of test accuracy studies
Source: Stat Methods Med Res. 2022 Jan 7;31(2):287–99. doi: 10.1177/09622802211065157 (PMC8829734; doi:10.1177/09622802211065157)
Supplement: sj-docx-1-smm-10.1177_09622802211065157 - Supplemental material for On estimating a constrained bivariate random effects model for meta-analysis of test accuracy studies [file sj-docx-1-smm-10.1177_09622802211065157.docx]

**Appendix**

|  |  | **Mean Bias(**${\hat{\boldsymbol{\sigma}}}_{\boldsymbol{a}}^{\mathbf{2}}$**)** | | **Mean Bias(**${\hat{\boldsymbol{\sigma}}}_{\boldsymbol{b}}^{\mathbf{2}}$**)** | | **Mean Bias(**$\hat{\boldsymbol{\rho}}$**)** | |
| --- | --- | --- | --- | --- | --- | --- | --- |
|  | { *k*, *τ^2^, ρ* } | **CBRM** | **BRM** | **CBRM** | **BRM** | **CBRM** | **BRM** |
| n = 50 | {10, 0.5, -0.5} | -0.0506 | -0.0580 | -0.0480 | -0.0535 | 0.0232 | 0.0245 |
| n = 100 | {10, 0.5, -0.5} | -0.0355 | -0.0574 | -0.0360 | -0.0584 | 0.0151 | 0.0232 |
| n = 250 | {10, 0.5, -0.5} | -0.0044 | -0.0572 | -0.0116 | -0.0515 | -0.0089 | 0.0169 |
| n = 500 | {10, 0.5, -0.5} | 0.0370 | -0.0604 | 0.0592 | -0.0546 | -0.0212 | 0.0253 |
| n = 1000 | {10, 0.5, -0.5} | 0.0861 | -0.0589 | 0.1151 | -0.0561 | -0.0421 | 0.0227 |
|  |  |  |  |  |  |  |  |
|  | { *τ^2^, ρ, n* } |  |  |  |  |  |  |
| k = 5 | {0.5, -0.5, 100} | -0.0642 | -0.0921 | -0.0558 | -0.0834 | 0.0625 | 0.0728 |
| k = 10 | {0.5, -0.5, 100} | -0.0355 | -0.0574 | -0.0360 | -0.0584 | 0.0151 | 0.0232 |
| k = 20 | {0.5, -0.5, 100} | -0.0137 | -0.0273 | -0.0107 | -0.0271 | -0.0026 | 0.0050 |
| k = 50 | {0.5, -0.5, 100} | 0.0003 | -0.0130 | 0.0050 | -0.0108 | -0.0086 | -0.0009 |
|  |  |  |  |  |  |  |  |
|  | { *k*, *ρ, n* } |  |  |  |  |  |  |
| $\tau^{2}$= 0.1 | {10,- 0.5, 100} | -0.0063 | -0.0084 | -0.0098 | -0.0105 | 0.0441 | 0.0427 |
| $\tau^{2}$= 0.5 | {10,- 0.5, 100} | -0.0355 | -0.0574 | -0.0360 | -0.0584 | 0.0151 | 0.0232 |
| $\tau^{2}$= 1.0 | {10,- 0.5, 100} | -0.0819 | -0.1429 | -0.0493 | -0.1151 | 0.0034 | 0.0201 |
| $\tau^{2}$= 1.5 | {10,- 0.5, 100} | -0.1437 | -0.2261 | -0.0551 | -0.1496 | 0.0054 | 0.0253 |
|  |  |  |  |  |  |  |  |
|  | { *k*, *τ^2^, n* } |  |  |  |  |  |  |
| $\rho$ = -0.1 | {10, 0.5, 100} | -0.0479 | -0.0588 | -0.0389 | -0.0515 | -0.0028 | 0.0031 |
| $\rho$ = -0.25 | {10, 0.5, 100} | -0.0403 | -0.0543 | -0.0344 | -0.0500 | 0.0001 | 0.0070 |
| $\rho$ = -0.5 | {10, 0.5, 100} | -0.0355 | -0.0574 | -0.0360 | -0.0584 | 0.0151 | 0.0232 |
| $\rho$ = -0.75 | {10, 0.5, 100} | -0.0322 | -0.0593 | -0.0343 | -0.0595 | 0.0249 | 0.0305 |
| $\rho$ = -0.9 | {10, 0.5, 100} | -0.0290 | -0.0640 | -0.0297 | -0.0685 | 0.0381 | 0.0362 |

Table A1: Mean bias of the estimated values of $\sigma_{a}^{2},\sigma_{b}^{2}$and $\rho$ for the CBRM and BRM based on10000 simulations for each scenario in the first stage of the simulations.

| Parameter |  | MSE $\hat{\sigma}_{a}^{2}$ | | MSE $\hat{\sigma}_{b}^{2}$ | | MSE $\hat{\rho}$ | |
| --- | --- | --- | --- | --- | --- | --- | --- |
|  | { *k*, *τ^2^, ρ* } | **CBRM** | **UBRM** | **CBRM** | **UBRM** | **CBRM** | **UBRM** |
| n = 50 | {10, 0.5, -0.5} | 0.0798 | 0.0740 | 0.0614 | 0.0581 | 0.0989 | 0.0976 |
| n = 100 | {10, 0.5, -0.5} | 0.1145 | 0.0768 | 0.0843 | 0.0593 | 0.0975 | 0.0965 |
| n = 250 | {10, 0.5, -0.5} | 0.1365 | 0.0878 | 0.1079 | 0.0591 | 0.0954 | 0.0949 |
| n = 500 | {10, 0.5, -0.5} | 0.1736 | 0.0753 | 0.1729 | 0.0611 | 0.0955 | 0.0967 |
| n = 1000 | {10, 0.5, -0.5} | 0.2368 | 0.0745 | 0.2463 | 0.0575 | 0.0963 | 0.0951 |
|  |  |  |  |  |  |  |  |
|  | { *τ^2^, ρ, n* } |  |  |  |  |  |  |
| k = 5 | {0.5, -0.5, 100} | 0.1861 | 0.1439 | 0.1527 | 0.1170 | 0.2190 | 0.2225 |
| k = 10 | {0.5, -0.5, 100} | 0.1145 | 0.0768 | 0.0843 | 0.0593 | 0.0975 | 0.0965 |
| k = 20 | {0.5, -0.5, 100} | 0.0493 | 0.0423 | 0.0394 | 0.0308 | 0.0459 | 0.0448 |
| k = 50 | {0.5, -0.5, 100} | 0.0250 | 0.0179 | 0.0216 | 0.0132 | 0.0190 | 0.0181 |
|  |  |  |  |  |  |  |  |
|  | { *k*, *ρ, n* } |  |  |  |  |  |  |
| $\tau^{2}$= 0.1 | {10,- 0.5, 100} | 0.0124 | 0.0098 | 0.0042 | 0.0045 | 0.1280 | 0.1228 |
| $\tau^{2}$= 0.5 | {10,- 0.5, 100} | 0.1145 | 0.0768 | 0.0843 | 0.0593 | 0.0975 | 0.0965 |
| $\tau^{2}$= 1.0 | {10,- 0.5, 100} | 0.2824 | 0.2386 | 0.2659 | 0.2119 | 0.0863 | 0.0868 |
| $\tau^{2}$= 1.5 | {10,- 0.5, 100} | 0.5693 | 0.5362 | 0.5606 | 0.5159 | 0.0852 | 0.0890 |
|  |  |  |  |  |  |  |  |
|  | { *k*, *τ^2^, n* } |  |  |  |  |  |  |
| $\rho$ = -0.1 | {10, 0.5, 100} | 0.0845 | 0.0783 | 0.0651 | 0.0581 | 0.1529 | 0.1495 |
| $\rho$ = -0.25 | {10, 0.5, 100} | 0.0993 | 0.0794 | 0.0701 | 0.0607 | 0.1367 | 0.1345 |
| $\rho$ = -0.5 | {10, 0.5, 100} | 0.1145 | 0.0768 | 0.0843 | 0.0593 | 0.0975 | 0.0965 |
| $\rho$ = -0.75 | {10, 0.5, 100} | 0.1097 | 0.0781 | 0.0792 | 0.0594 | 0.0438 | 0.0453 |
| $\rho$ = -0.9 | {10, 0.5, 100} | 0.1674 | 0.0832 | 0.1564 | 0.0699 | 0.0283 | 0.0258 |

Table A2: Mean squared error of the estimated values of $\sigma_{a}^{2},\sigma_{b}^{2}$and $\rho$ and ρ for the CBRM and BRM based on10000 simulations for each for each scenario in the first stage of the simulations.

|  |  |  |  | **Mean Bias(**${\hat{\boldsymbol{\sigma}}}_{\boldsymbol{a}}^{\mathbf{2}}$**)** | | **Mean Bias(**${\hat{\boldsymbol{\sigma}}}_{\boldsymbol{b}}^{\mathbf{2}}$**)** | | **Mean Bias(**$\hat{\boldsymbol{\rho}}$**)** | |
| --- | --- | --- | --- | --- | --- | --- | --- | --- | --- |
| $n$ | $k$ | $\tau^{2}$ | $\rho$ | **CBRM** | **BRM** | **CBRM** | **BRM** | **CBRM** | **BRM** |
| 50 | 5 | 0.1 | -0.1 | -0.0065 | -0.0102 | 0.0081 | -0.0104 | 0.0236 | 0.0221 |
| 50 | 5 | 0.1 | -0.9 | 0.0074 | 0.0036 | -0.0047 | -0.0070 | 0.1191 | 0.1096 |
| 50 | 5 | 1.5 | -0.1 | -0.2719 | -0.2974 | -0.1776 | -0.2028 | 0.0125 | 0.0127 |
| 50 | 5 | 1.5 | -0.9 | -0.2026 | -0.2951 | -0.1945 | -0.2804 | 0.1025 | 0.1041 |
| 50 | 50 | 0.1 | -0.1 | -0.0036 | -0.0040 | -0.0021 | -0.0022 | -0.0078 | -0.0078 |
| 50 | 50 | 0.1 | -0.9 | 0.0026 | -0.0012 | -0.0012 | -0.0025 | 0.0226 | 0.0204 |
| 50 | 50 | 1.5 | -0.1 | -0.1044 | -0.1364 | -0.0349 | -0.0532 | -0.0033 | 0.0011 |
| 50 | 50 | 1.5 | -0.9 | 0.0171 | -0.0448 | 0.0232 | -0.0385 | 0.0034 | 0.0048 |
|  |  |  |  |  |  |  |  |  |  |
| 1000 | 5 | 0.1 | -0.1 | 0.0168 | -0.0068 | 0.0091 | -0.0099 | -0.0024 | 0.0192 |
| 1000 | 5 | 0.1 | -0.9 | 0.0263 | -0.0012 | 0.0196 | -0.0092 | 0.1242 | 0.1132 |
| 1000 | 5 | 1.5 | -0.1 | -0.0702 | -0.2995 | 0.0599 | -0.2310 | -0.0892 | 0.0096 |
| 1000 | 5 | 1.5 | -0.9 | 0.2294 | -0.2932 | 0.2558 | -0.2812 | 0.0962 | 0.1030 |
| 1000 | 50 | 0.1 | -0.1 | 0.0028 | -0.0040 | 0.0055 | -0.0024 | -0.0261 | -0.0031 |
| 1000 | 50 | 0.1 | -0.9 | 0.0229 | 0.0011 | 0.0142 | -0.0037 | 0.0235 | 0.0229 |
| 1000 | 50 | 1.5 | -0.1 | 0.0474 | -0.1375 | 0.1291 | -0.0556 | -0.0750 | 0.0019 |
| 1000 | 50 | 1.5 | -0.9 | 0.6017 | -0.0383 | 0.6192 | -0.0395 | -0.0107 | 0.0044 |

Table A3: Mean bias of the estimated values of $\sigma_{a}^{2},\sigma_{b}^{2}$and $\rho$ for the CBRM and BRM based on10000 simulations for each scenario in the second stage of the simulations.

|  |  |  |  | MSE($\hat{\sigma}_{a}^{2}$) | | MSE($\hat{\sigma}_{b}^{2}$) | | MSE($\hat{\rho}$) | |
| --- | --- | --- | --- | --- | --- | --- | --- | --- | --- |
|  |  |  |  |  |  |  |  |  |  |
| $n$ | $k$ | $\tau^{2}$ | $\rho$ | **CBRM** | **BRM** | **CBRM** | **BRM** | **CBRM** | **BRM** |
| 50 | 5 | 0.1 | -0.1 | 0.0293 | 0.0148 | 0.0292 | 0.0188 | 0.2956 | 0.2906 |
| 50 | 5 | 0.1 | -0.9 | 0.0768 | 0.0615 | 0.0419 | 0.0373 | 0.1072 | 0.0990 |
| 50 | 5 | 1.5 | -0.1 | 1.0654 | 1.0550 | 1.1628 | 1.1691 | 0.2733 | 0.2737 |
| 50 | 5 | 1.5 | -0.9 | 1.5412 | 1.3537 | 1.4468 | 1.3031 | 0.1036 | 0.1899 |
| 50 | 50 | 0.1 | -0.1 | 0.0024 | 0.0019 | 0.0021 | 0.0020 | 0.0505 | 0.0487 |
| 50 | 50 | 0.1 | -0.9 | 0.0207 | 0.0084 | 0.0110 | 0.0101 | 0.0092 | 0.0092 |
| 50 | 50 | 1.5 | -0.1 | 0.1419 | 0.1541 | 0.1133 | 0.1126 | 0.0242 | 0.0234 |
| 50 | 50 | 1.5 | -0.9 | 0.2203 | 0.1381 | 0.2046 | 0.1228 | 0.0025 | 0.0023 |
|  |  |  |  |  |  |  |  |  |  |
| 1000 | 5 | 0.1 | -0.1 | 0.0864 | 0.0188 | 0.0494 | 0.0124 | 0.3045 | 0.2861 |
| 1000 | 5 | 0.1 | -0.9 | 0.0719 | 0.0403 | 0.0771 | 0.0415 | 0.1091 | 0.1112 |
| 1000 | 5 | 1.5 | -0.1 | 1.2611 | 1.1062 | 1.3105 | 1.1167 | 0.2742 | 0.2823 |
| 1000 | 5 | 1.5 | -0.9 | 1.0945 | 1.3843 | 1.0834 | 1.2623 | 0.1027 | 0.1781 |
| 1000 | 50 | 0.1 | -0.1 | 0.0052 | 0.0019 | 0.0024 | 0.0010 | 0.0578 | 0.0489 |
| 1000 | 50 | 0.1 | -0.9 | 0.0648 | 0.0365 | 0.0211 | 0.0032 | 0.0118 | 0.0080 |
| 1000 | 50 | 1.5 | -0.1 | 0.1890 | 0.1572 | 0.2107 | 0.1129 | 0.0404 | 0.0231 |
| 1000 | 50 | 1.5 | -0.9 | 1.6610 | 0.1290 | 1.7011 | 0.1150 | 0.0046 | 0.0026 |

Table A4: Mean squared error of the estimated values of $\sigma_{a}^{2},\sigma_{b}^{2}$and $\rho$ and ρ for the CBRM and BRM based on10000 simulations for each scenario in the second stage of the simulations.

Figure A1: Distribution of Euclidean distances for the unconstrained and constrained models at different values of k. The boxes are bounded by the lower and upper quartiles and the caps to the whiskers represent the minimum and maximum for the data. The dashed horizontal lines represent the 99.5 percentiles.

Figure A2: Distribution of Euclidean distances for the unconstrained and constrained models at different values of n. The boxes are bounded by the lower and upper quartiles and the caps to the whiskers represent the minimum and maximum for the data. The dashed horizontal lines represent the 99.5 percentiles.

Figure A3: Distribution of Euclidean distances for the unconstrained and constrained models at different values of $\tau^{2}$. The boxes are bounded by the lower and upper quartiles and the caps to the whiskers represent the minimum and maximum for the data. The dashed horizontal lines represent the 99.5 percentiles.

Figure A4: Distribution of Euclidean distances for the unconstrained and constrained models at different values of $\rho$. The boxes are bounded by the lower and upper quartiles and the caps to the whiskers represent the minimum and maximum for the data. The dashed horizontal lines represent the 99.5 percentiles.

Figure A5: Distribution of Euclidean distances for the unconstrained and constrained models at the shown values of k, n, $\tau^{2}$ and $\rho$. The boxes are bounded by the lower and upper quartiles and the caps to the whiskers represent the minimum and maximum for the data. The dashed horizontal lines represent the 99.5 percentiles.

 Figure A6: Distribution of Euclidean distances for the unconstrained and constrained models at the shown values of k, n, $\tau^{2}$ and $\rho$. The boxes are bounded by the lower and upper quartiles and the caps to the whiskers represent the minimum and maximum for the data. The dashed horizontal lines represent the 99.5 percentiles.

Figure A7: Distribution of Euclidean distances for the unconstrained and constrained models at the shown values of k, n, $\tau^{2}$ and $\rho$. The boxes are bounded by the lower and upper quartiles and the caps to the whiskers represent the minimum and maximum for the data. The dashed horizontal lines represent the 99.5 percentiles.

 Figure A8: Distribution of Euclidean distances for the unconstrained and constrained models at the shown values of k, n, $\tau^{2}$ and $\rho$. The boxes are bounded by the lower and upper quartiles and the caps to the whiskers represent the minimum and maximum for the data. The dashed horizontal lines represent the 99.5 percentiles.
